# Housing Accessibility at Home and Rehabilitation Outcomes After a Stroke: An Explorative Study

**DOI:** 10.1177/19375867231178313

**Published:** 2023-06-07

**Authors:** Marie Elf, Björn Slaug, Charlotte Ytterberg, Ann Heylighen, Maya Kylén

**Affiliations:** 1 School of Health and Welfare, Dalarna University, Falun, Sweden; 2 Department of Health Sciences, Lund University, Sweden; 3 Department of Neurobiology, Care Sciences and Society, Karolinska Institutet, Huddinge, Sweden; 4 Women’s Health and Allied Health Professionals Theme, Karolinska University Hospital, Stockholm, Sweden; 5 Research[x]Design, Department of Architecture, KU Leuven, Belgium

**Keywords:** stroke, rehabilitation, person–environment fit, built environment, shared–decision making

## Abstract

**Purpose:**

To explore if aspects of the physical home environment are related to rehabilitation outcomes among community-living persons poststroke.

**Background:**

Research demonstrates that healthcare environments are important for high-quality care and that the design of the physical environment is associated with improved rehabilitation outcomes. However, relevant research focusing on outpatient care settings, such as the home, is sparse.

**Methods:**

In this cross-sectional study, data on rehabilitation outcomes, physical environmental barriers, and housing accessibility problems were collected during home visits of participants (*N* = 34), 3 months poststroke. Data were analyzed with descriptive statistics and correlation analysis.

**Results:**

Few participants had adapted their homes, and the relevance of the physical environment was not always discussed with the patient during discharge from the hospital. Accessibility problems were associated with suboptimal rehabilitation outcomes such as worse perceived health and recovery after stroke. Activities most restricted by barriers in the home concerned hand and arm use. Participants who reported one or more falls at home tended to live in houses with more accessibility problems. Perceived supportive home environments were associated with more accessible dwellings.

**Conclusions:**

Many face problems adapting their home environments poststroke, and our findings highlight unmet needs that should be considered in the rehabilitation practice. These findings could be used by architectural planners and health practitioners for more effective housing planning and inclusive environments.

## Introduction

This study focuses on persons who have had a stroke and are rehabilitating at home. While there is research demonstrating that healthcare environments are important for high-quality care and that the design of the physical environment is associated with improved health outcomes (Edvardsson et al., 2008; Henriksen et al., 2007; Lipson-Smith et al., 2021; Ulrich et al., 2008), relevant research focusing on outpatient care settings, such as the home, is sparse. Today, hospital stays are short and patients are discharged to home “quicker and sicker” which makes the physical home environment central to supporting poststroke rehabilitation (Abdi et al., 2019; Marcheschi et al., 2018; Schneidert et al., 2003). However, research has revealed that when rehabilitation after a stroke takes place at home, the physical environment is only integrated into the rehabilitation process to a limited extent (Kylén et al., 2021).

***Today, hospital stays are short and patients are discharged to home “quicker and sicker” which makes the physical home environment central to supporting poststroke rehabilitation***

As people have different functional abilities poststroke, which are displayed during rehabilitation, the same environment can facilitate an activity for one person and hinder an activity for another. This notion underscores the importance of accessibility, which can be defined as the relationship between the individual’s functional capacity and the demands generated by the environmental design (Iwarsson & Ståhl, 2003). The concept is based on the ecological theory of aging, also known as the competence-press or person–environment fit model (Lawton & Nahemow, 1973) which posits that individuals with lower personal competencies are more susceptible to environmental press while those with higher competencies can better handle greater environmental press. Hence, accessibility problems arise when the demands of the environment surpass the functional capacity of a person; for instance, uneven flooring may create severe problems for people with poor balance.

To consider accessibility during the rehabilitation is important because after a stroke, more than 30% of individuals experience limitations in activities of daily living (ADL; Blomgren et al., 2019), and the percentage increases over time (Gil-Salcedo et al., 2022), making stroke the main cause of long-term disability worldwide. However, while accessibility problems in the home are related to ADL dependence among people 67–84 years old in the general population (Iwarsson, 2005), as well as among persons with Parkinson’s disease (Gefenaite et al., 2020), similar research in the context of stroke rehabilitation is lacking.

Research has shown that living in an accessible home supports independence, health, and well-being for older adults in the general population (Wahl et al., 2009) and environmental barriers are associated with disability-related outcomes in older age (Rantakokko et al., 2013). In Sweden, despite high housing standards, accessibility problems are common and become more severe for those with more pronounced functional limitations (e.g., being dependent on a mobility aid or device; Granbom et al., 2016). This is a concern as falls at home are common in the older adult population, in general, and in persons with complex health conditions (e.g., stroke), in particular (Siqueira et al., 2007). In addition to adverse health outcomes (e.g., fractures) for the individual, the high prevalence of falls is associated with substantial economic costs for society (Stevens et al., 2006). Thus, to prevent falls, it is essential to consider the physical home environment and target persons’ needs.

Being at home in a familiar place can support the recovery process. It creates opportunities for rehabilitation activities in a natural environment rather than a in a controlled environment such as the hospital. Thus, the home environment allows problem-solving and planning in a more meaningful context. Nevertheless, after a stroke, many feel abandoned and not adequately supported; therefore, coming home may be a stressful event (Lindblom et al., 2020; Meijering et al., 2016). This is a challenge not only for the patient but also for caregivers (Garnett et al., 2022) and practitioners who are expected to provide care and work in various housing situations. Moreover, a home is closely linked to a person’s identity (Kylén et al., 2017), which practitioners must consider while providing technical aid or making changes to the home environment. Such a situation makes it important for practitioners to involve the person as an active partner in rehabilitation planning and focus on the patient’s needs and resources as well as the environment (Yun & Choi, 2019). Thus, feeling involved in one’s rehabilitation is an essential aspect of achieving positive health outcomes, particularly for individuals with complex health conditions, but research has shown that this group feels disconnected from their care and rehabilitation (Kylén at al., 2021; Vårdanalys, 2018, p. 8) which is a concern that urgently calls for more attention in research and clinical practice.

In this study, we use the International Classification of Functioning, Disability and Health (ICF; Schneidert et al., 2003) as a framework to understand the importance of the environment in stroke rehabilitation practice. The ICF underscores the significance of the environment for people’s health and well-being, which is perceived as an outcome of the complex dynamic interaction between functioning (i.e., bodily function/activity/participation) and contextual factors (i.e., personal, and environmental factors). The framework explains how the physical environment for stroke rehabilitation can either be perceived as a facilitator or a barrier to a person’s level of functioning. The physical environment is described as both the immediate setting of the home and its surroundings (i.e., neighborhood). The ICF clearly supports empirical research findings demonstrating that the dynamic relationship between the person and their physical and perceived environment is important for health and well-being (Kylén et al., 2017; Oswald et al., 2007; Whal et al., 2009, 2012). To improve rehabilitation outcomes, it is important to consider not just a person’s functional status or social factors but also the relationship between all aspects of their life situation and physical environment. This approach aligns with the main principles of the ICF.

The most established home-based stroke rehabilitation model is Early Supported Discharge (ESD; Langhorne et al., 2017). This multidisciplinary team intervention facilitates discharge from the hospital and rehabilitation at home at an intensity comparable to that provided in hospitals (Langhorne et al., 2017). Research has shown that ESD can reduce the length of hospital stay and the risk of dependency of stroke survivors (Langhorne et al., 2005). However, despite these promising results, the model is not fully implemented in practice (SBU, 2015). In addition, ESD guidelines do not include the physical environment as an important aspect to consider in the rehabilitation process (Langhorne et al., 2017). In sum, there is evidence that home rehabilitation can be a positive and viable option for people with stroke. Still, we know very little about the mechanisms that affect patient outcomes, especially the physical environmental factors that may play a role in a person’s rehabilitation process (Marcheschi et al., 2018).

***we know very little about the mechanisms that affect patient outcomes, especially the physical environmental factors that may play a role in a person’s rehabilitation process***

Therefore, focusing on a sample of community-living persons poststroke, the aims of this study were to (1) explore if aspects of the physical home environment are related to rehabilitation outcomes, (2) explore which activities were most restricted owing to accessibility problems at home, and (3) describe the persons’ degree of partaking in the rehabilitation planning, with a specific focus on the physical environment.

## Method

### Study Context and Participants

This explorative cross-sectional study is a part of a larger project named Rehabilitation and Architecture (REARCH), in which we study the interaction between the physical environment and recovery among people with stroke who are rehabilitating at home, see Kylén et al. (2019) for a study protocol. We recruited 34 patients with stroke from three stroke units in the south of Sweden. Eligible persons were identified and informed about the study by ESD team members (e.g., occupational therapists, physiotherapists) working at participating hospitals. To be included, patients needed to have had a mild to moderate stroke, according to the Barthel Index (Govan et al., 2009), have been discharged directly to their homes and be able to communicate and formulate answers to questions in an interview. Patients who were willing to participate received an informed consent form and were then contacted by a member of the research team to schedule a time for data collection.

### Ethical Considerations

The study was conducted in accordance with the Helsinki Declaration and approved by the Swedish Ethical Review Authority (2015/389). All participants provided oral and written informed consent.

### Data Collection Procedures

Data were collected between August 2019 and March 2020 during home visits at a time decided by the participants. The data were collected by the last author Maya Kylén, who has extensive experience in using the Housing Enabler (HE) instrument (described below). The participants had the opportunity to ask questions and be assisted with writing if needed due to, for example, weakness in the hand.

#### Environmental barriers and housing accessibility problems

Data on environmental barriers and accessibility problems were collected with the HE instrument, which is based on extensive research (Iwarsson et al., 2012) and has proved to be reliable and valid (Helle et al., 2010). The instrument is based on Swedish national standards for housing design and is administrated in three steps.

- In Step 1, functional limitations (12 items, e.g., visual impairment, poor balance, reduced fine motor skill, etc.) and dependence on mobility devices (two items) are dichotomously assessed (present/not present) during an interview and observation. Step 1 provides a profile of functional limitations and renders a sum score of functional limitations (range 0–12) and dependence on mobility devices (range 0–2). Collected data in Step 1 (12 + 2 items) was also used for the variable Functional profile as an indicator of a person’s overall physical health.
- Step 2 includes an observation and a dichotomous assessment (checklist with present/not-present) of 161 environmental barriers that can be divided in three subdomains: 87 at home (e.g., no grab bar at shower/bath and/or toilet), 46 at the entrance of the building (e.g., high threshold/level difference/step), and 28 in the immediate surrounding environment (e.g., refuse room/refuse bin can only be reached via steps or other differences in level). Step 2 provides a sum score of environmental barriers (range 0–161/or divided in the three subdomains) as well as a detailed description of present environmental barriers.
- Based on the results from Steps 1 and 2, Step 3 involves a person–environment fit analysis. By juxtaposing the personal and environmental components, a total score is calculated—this quantifies the magnitude of accessibility problems in a particular case. A higher score indicates more accessibility problems. In cases with no functional limitations/dependence on mobility devices present, the total score is always 0, regardless of the environmental barriers.

#### Housing adaptations

Housing adaptations were self-reported and observed during Step 2 using the HE described above. During an initial interview, participant were asked if they had any housing adaptations made in the residence and if so where they were located (e.g., entrance, kitchen, bathroom).

#### Activities restricted owing to accessibility problems at home

To examine potential constraining effects that physical environmental barriers at home may have on activities performed, we used a previously published typology of person–environment fit constellations (Slaug et al., 2015). The typology links each environmental barrier included in the HE with activity in terms of the ICF. For instance, an environmental barrier like the lack of handrails along a staircase at home may restrict movement for a person with balance problems; an environmental barrier like too high/low seats may hinder a person with difficulties bending and kneeling from sitting. In such instances, the lack of handrails is linked to ICF activity: moving around and too high/low seats to changing basic body position. The typology uses the ICF levels of block and category.

### Rehabilitation Outcomes

In this study, rehabilitation outcomes were measured with self-reported instruments and study specific questions including falls, perceived health, perceived impact of stroke, self-efficacy and decision making.

#### Perceived health

Participants’ perceived health was measured with the visual analogue scale from the EuroQual 5 (EQ-5D), ranging from 0 (*worst imaginable health*) to 100 (*best imaginable health*). The EQ-5D is a self-reported scale and has proved to be valid and sensitive to change (Golicki et al., 2015).

#### Perceived impact of stroke

Perceived impact of stroke was measured with one item (ranging from 0 = *no recovery* to 100 = *maximum recovery*) from the Stroke Impact Scale, Version 2.0, a self-report instrument that evaluates health-related quality of life and disability after stroke (Duncan et al., 1999).

#### Self-efficacy

Self-efficacy was measured with the 10-item General Self-Efficacy (GSE) Scale assessing a person’s ability to cope with a variety of difficult demands in life (Schwarzer & Jerusalem, 1995). Individuals rate their scores on a 4-point Likert-type scale ranging from 1 = *not at all true* to 4 = *exactly true*, where higher scores (sum score range: min = 10, max = 40) indicate a greater sense of GSE. We used the validated Swedish version of the GSE (Carlstedt et al., 2015; Löve et al., 2012).

#### Falls

Falls were self-reported. Participants were asked if and how many times they had fallen between hospital discharge and the time of the data collection.

#### Decision making and possibilities of discussing the physical environment with healthcare staff

We used four project-specific questions to explore the patient’s participation in healthcare and the possibilities of discussing the home environment at the hospital and at home: (1) Overall, to what degree do you feel that you have been involved in the planning of your healthcare/treatment/rehabilitation?; (2) At the time of discharge from the hospital, to what degree were you given the opportunity to discuss the home environment while planning your continued care/treatment/rehabilitation at home?; (3) At the time of hospital discharge, to what degree did the staff ask if there were any obstacles in your home?; and (4) After you came home, to what degree did the ESD rehabilitation staff talk about possible obstacles in your home? In addition, we asked a question about perceived environmental support: To what degree do you think your home environment supports you, or constitutes an obstacle for you, in terms of the everyday activities you want to and must perform? Responses were rated on a 5-point Likert-type scale ranging from 1 = *not at all* to 5 = *very much*. For the last question, responses were rated on a scale ranging from 1 = *a major obstacle* to 5 = *a lot of support*.

### Data Analyses

Descriptive statistics were used to present sample characteristics, health and rehabilitation variables, as well as environmental barriers and accessibility problem scores. For each of the 161 environmental barriers in the HE, an average accessibility problem score was computed, ranging from 0 to 35. To produce a ranking list of the environmental barriers according to the degree of accessibility problems they generated, they were sorted in descending order based on the average scores; the 15 top scores were listed. To disentangle the accessibility problems in terms of restricted activities, we calculated the relative proportions of the accessibility problem score by the linked ICF activities. To analyze associations between housing accessibility and rehabilitation outcomes, we utilized the Spearman correlation analysis. The SAS Software, SAS Institute Inc., Cary, NC, Version 9.4 was used for all analyses. *p* Values < .05 were considered statistically significant.

## Results

### Sample Characteristics

There were more men (*n* = 21) than women (*n* = 13) participating in the study; the average age of the participants was 72 years ranging from 34 to 90. Of the 34 participants, approximately half were living alone (*n* = 18) and the majority lived in a multifamily type of dwelling (*n* = 19). The length of stay in the present home ranged from 19.3 to 27.5 years among the 34 participants. Six persons had implemented housing adaptations and 50% of the study sample (*n* = 17, 11 men, 6 women) were reliant on a walking aid. The median number of days since stroke onset was 84 (59–101), and the participants reported between two and six functional limitations. See Table 1 for sample characteristics.

**Table 1.** Sample Characteristics.

	Men (*n* = 21)	Women (*n* = 13)	Total (*n* = 34)
Characteristics			
Age, mean (*SD*)	72.0 (10.9)	71.5 (15.0)	71.9 (12.4)
Days since stroke, median (q1–q3)	84 (70–97)	85 (55–106)^a^	84 (59–101) ^a^
Living situation			
Cohabiting, *n* (column %)	12 (57)	4 (31)	16 (47)
Single living, *n* (column %)	9 (43)	9 (69)	18 (53)
Type of housing			
Multifamily building, *n* (column %)	12 (57)	7 (54)	19 (56)
Single-family building, *n* (column %)	8 (38)	5 (38)	13 (38)
Senior housing, *n* (column %)	1 (5)	1 (8)	2 (6)
Years in home, mean (*SD*)	19.3 (11.8)	27.5 (17.5)	22.4 (16.8)
Presence of housing adaptations, *n* (%)	2 (10)	4 (31)	6 (19)
Functional profile ^a,b^			
Number of functional limitations, median (q1–q3)^c^	6 (2–6)	6 (4–6)	6 (4–6)
Dependence on mobility devices			
Reliance on walking aids, *n* (%)	11 (52)	6 (46)	17 (50)

^a^ Missing value, *n* = 1. ^b^ Housing Enabler instrument (Iwarsson et al., 2012). ^c^ Min-max: 0–12.

### Environmental Barriers and Accessibility Problems

Environmental barriers were identified in all dwellings assessed (Table 2). The median number of barriers present was 71 in multifamily dwellings, 71 in senior housing, and 62 in single-family dwellings. In terms of accessibility problems, the median score was 288, with most problems identified indoors, regardless of the type of dwelling. High accessibility problem scores at the entrances were more frequent in senior housing and multifamily dwellings, while high accessibility problem scores for the exterior surroundings were more common in multifamily dwellings (see Table 2 for details).

***Environmental barriers were identified in all dwellings assessed***

**Table 2.** Number of Environmental Barriers in Relation to Type of Housing and Magnitude of Accessibility Problems.

Variable	Multifamily Dwellings (*n* = 18)	Single-Family Dwellings (*n* = 13)	Senior Housing (*n* = 2)	All Dwellings (*N* = 33)
Environmental barriers/housing section ^a^ (min-max: 0–161)				
Exterior surroundings, median (q1–q3)	15 (12–16)	9 (8–11)	11 (7–15)	12 (9–15)
Entrances, median (q1–q3)	13 (11–19)	4 (3–6)	17 (10–23)	10 (5–13)
Indoors, median (q1–q3)	42 (39–44)	48 (43–52)	44 (34–53)	43 (40–49)
Total, median (q1–q3)	71 (67–77)	62 (60–63)	71 (51–91)	68 (60–74)
Accessibility problem score/housing section ^a^ (min-max: 0–1,844)				
Exterior surroundings, median (q1–q3)	89 (67–106)	57 (13–69)	63 (55–70)	70 (46–96)
Entrances, median (q1–q3)	66 (51–120)	15 (6–24)	67 (54–79)	52 (21–58)
Indoors, median (q1–q3)	156 (140–168)	92 (66–245)	104 (84–124)	153 (84–171)
Total, median (q1–q3)	321 (285–385)	156 (78–338)	233 (193–273)	288 (156–356)

*Note*. *N* = 33.

^a^ Housing Enabler instrument (Iwarsson et al., 2012).

Among the top 15 environmental barriers generating high accessibility problem scores (min-max score 0–35), six were identified indoors, five in the exterior surroundings, and four at the entrances (Table 3). Indoors, “wall-mounted cupboards and shelves placed high in the kitchen” (11.9) and “no grab bar at shower/bath and/or toilet in the bathroom” (10.6) generated the most accessibility problems. In the exterior surroundings, “difficulty to reach the refuse bin” (8.5) and “to reach the letter box” (8.3) were ranked highest. “Stairs as the only route (i.e., no elevator)” (7.4) and “high thresholds and/or steps” (5.7) generated the most accessibility problems at the entrances (see Table 3 for details).

**Table 3.** The 15 Environmental Barriers Generating Most Accessibility Problems.

Housing Section	Environmental Barrier	Rank (Score) ^a^	Prevalence,%
Indoor	Wall-mounted cupboards and shelves placed high	1 (11.9)	73
Indoor	No grab bar at shower/bath and/or toilet	2 (10.6)	88
Exterior	Refuse bin difficult to reach	3 (8.5)	85
Exterior	Letter box difficult to reach	4 (8.3)	85
Entrance	Stairs the only route (no lift/ramp)	5 (7.4)	41
Indoor	Storage areas can be reached only via stairs/threshold or other difference in level	6 (6.0)	91
Entrance	High thresholds and/or steps at the entrance	7 (5.7)	91
Entrance	High threshold/level difference/step	7 (5.7)	91
Exterior	Poorly drained paths and roadways	9 (5.2)	70
Indoor	Steps/thresholds/differences in level between rooms/floor spaces	9 (5.2)	79
Indoor	Insufficient maneuvering spaces in relation to movable furnishings	11 (5.0)	91
Entrance	Heavy doors without automatic opening.	12 (4.2)	36
Indoor	Laundry room can be reached only via stairs/threshold or other difference in level	13 (4.1)	62
Exterior	Irregular/uneven surface (irregular surfacing, joins, sloping sections cracks, holes)	14 (4.0)	91
Exterior	Refuse room/refuse bin can only be reached via steps or other differences in level	15 (3.9)	61

*Note*. *N* = 33.

^a^ Accessibility problem score, Housing Enabler instrument. Min-max score: 0–35.

### Health and Rehabilitation Variables in the Study Sample

More than half of the study participants (59%) had fallen at home at least once since their discharge from the hospital. Women reported better perceived health than men, with the median score being 80, compared to 70 among the men. Perceived recovery after stroke varied among the participants between 45 and 85 and was slightly higher among the women. The vast majority of the participants stated that they lived in a home that supported their everyday activities. Regarding GSE, the median score was the same for men and women with a somewhat larger range among the men (Table 4).

***More than half of the study participants (59%) had fallen at home at least once since their discharge from the hospital***

**Table 4.** Health and Rehabilitation Variables in the Study Sample.

Variables	Men (*n* = 21)	Women (*n* = 13)	Total (*N* = 34)
Falls, *n* (%)	14 (67)	6 (46)	20 (59)
Perceived health, median (q1–q3) ^a^	70 (50–85)	80 (70–85)	73 (60–85)
Perceived recovery after stroke, median (q1–q3) ^a^	65 (45–85)	70 (40–80)	70 (45–85)
Perceived environmental support at home, median (q1–q3)^b^	5 (4–5)	5 (4–5)	5 (4–5)
Decision making in care and rehabilitation planning, median (q1–q3)^b^	3 (1–4)	3 (3–4)	3 (1–4)
Possibility to discuss the environment at hospital discharge, median (q1–q3)^b^	2 (1–5)	2 (1–4)	2 (1–4)
Possibility to discuss barriers at home at hospital discharge, median (q1–q3)^b^	3 (1–5)	2 (1–3)	2 (1–5)
Possibility to discuss barriers with Early Supported Discharge team at home, median (q1–q3)^b^	5 (2–5)	4 (2–4)	4 (2–5)
General self-efficacy, median (q1–q3)^c^	30 (28–35)	30 (27–32)	30 (28–35)

^a^ Min-max score: 0–100. ^b^ Min-max score: 1–5. ^c^ Min-max score: 10–40.

### Accessibility Problems and Rehabilitation Outcomes

As presented in Table 5, a high accessibility problem score demonstrated a moderate but significant negative relationship with perceived health (*r* = .43, *p* = .012), recovery after stroke (*r* = −.40, *p* = .022), GSE (*r* = −.42, *p* = .017), and perceived environmental support in the home (*r* = −.35, *p* = .043). This suggests that overall, living in a highly inaccessible building was associated with worse rehabilitations outcomes in the study sample. In terms of the different housing sections, a high accessibility problem score indoors was negatively correlated with perceived health (*r* = −.42, *p* = .016) and GSE (*r* = −.45, *p* = .009). While these correlations were moderate, the correlation between high scores indoors and recovery after stroke was low (*r* = −.36, *p* = .042). The negative correlations between perceived health (*r* = −.43, *p* = 0.012), recovery after stroke (*r* = −.44, *p* = 0.010), and accessibility problems at the entrances were moderate. Accessibility problems in the close exterior surroundings were only negatively correlated to GSE (*r* = −.39, *p* = .026) and perceived environmental support in the home (*r* = −.50, *p* = .003; Table 5). In addition, although not significant (*p* = .277), participants who reported one or more falls at home (*n* = 20) tended to live in houses with more accessibility problems (median = 330, interquartile range = 236) than those who had not fallen (*n* = 13, median = 273, interquartile range = 173; not in table).

***living in a highly inaccessible building was associated with worse rehabilitations outcomes in the study sample.***

**Table 5.** Correlation Between Housing Accessibility (Number of Environmental Barriers and Accessibility Problem Score) and Rehabilitation Outcomes.

Variables	Perceived Health	Recovery After Stroke	General Self-Efficacy	Perceived Environmental Support
	Spearman correlation coefficient (*p* value)			
Accessibility problem score, close exterior surroundings	−.31 (.082)	.28 (.111)	**−.39 (.026)**	**−.50 (.003)**
Accessibility problem score, entrances	**−.43 (.012)**	**−.44 (0.010)**	.29 (.105)	−.26 (.136)
Accessibility problem score, indoor	**−.42 (.016)**	**−.36 (.042)**	**−.45 (.009)**	−.34 (.054)
Accessibility problem score, total	**−.43 (.012)**	**−.40 (.022)**	**−.42 (.017)**	**−.35 (.043)**

*Note*. Significant correlations are bolded. High accessibility problem scores mean more problems.

### Activities Restricted by Accessibility Problems in the Home

The activities most restricted by environmental barriers in the home concerned hand and arm use, that is, reaching, pulling, and pushing objects. Such barriers (e.g., accessing wall-mounted cupboards and shelves placed high) contributed to 24.9% of the total accessibility problem score. Additionally, activities most restricted by environmental barriers in the home were walking (on different surfaces and around objects) and maintaining body position (sitting or standing), which contributed to the total accessibility problem score with 23% and 12.4%, respectively. For further details, see Table 6.

***The activities most restricted by environmental barriers in the home concerned hand and arm use***

**Table 6.** Activities in Terms of International Classification of Functioning, Disability and Health (ICF), Constrained by Accessibility Problems in the Home.

ICF Code	ICF Activity Constrained by Accessibility Problems ^a^	Contribution to Accessibility Problem Score (%)
D445	Hand and arm use (i.e., reaching, pulling, pushing)	24.9
D450	Walking (i.e., on different surfaces, around obstacles)	23.0
D415	Maintaining body position (i.e., sitting, standing position)	12.4
D460	Moving around in different locations (i.e., inside/outside home)	8.7
D440	Fine hand use (i.e., grasping, manipulating, turning, twisting)	8.1
D455	Moving around (i.e., climbing stairs)	7.6
D410	Changing basic body position (i.e., sitting down, standing up)	5.0
D465	Moving around using equipment (i.e., mobility devices)	4.8
D110	Watching (i.e., purposeful visual experience)	3.0
D179	Applying knowledge (i.e., interpreting information)	1.4
D115	Listening (i.e., purposeful audio experience)	0.8
D430	Lifting and carrying objects (i.e., by hands)	0.3
D420	Transferring oneself (i.e., while sitting)	<0.1
Total		100.0

^a^ According to a typology of P-E fit constellations (Slaug et al., 2015).

### Decision Making and Possibilities of Discussing the Physical Environment With the Healthcare Staff

More than half of the study participants reported to be moderately involved, and 35% responded not at all or little. At the time of discharge from the hospital, few were given the opportunity to discuss the home environment, although men, to a larger extent than women, were asked about possible environmental barriers in their home. In comparison to experiences gained at the hospital, the participants as a group had more opportunities to discuss possible barriers in the home environment with the ESD staff (higher among men; see Table 4 for details).

***More than half of the study participants reported to be moderately involved, and 35% responded not at all or little***

## Discussion

This explorative study suggests that the physical home environment is important to consider in poststroke rehabilitation as it may negatively affect rehabilitation outcomes. Overall, the findings highlight unmet needs. Many participants faced accessibility problems, but very few had housing adaptation, and the physical home environment was not always reported to be discussed with the patient when he/she was discharged from the hospital. While hitherto seldom reported in stroke research, such knowledge is important as rehabilitation increasingly takes place in patients’ home environments and not in the hospital.

The finding that environmental barriers were more common in multifamily dwellings than single-family dwellings, as reflected by other studies (Kylén et al., 2014; SOU 2015), is not surprising. However, considering that many people who are discharged from the hospital after a stroke live in multifamily dwellings, efforts should always be made to understand each person’s home conditions prior to discharge. As in other studies (e.g., Siqueira et al., 2007), we found a high prevalence of falls after discharge (59%), which suggests that paying attention to environmental barriers could potentially prevent falls. That is, a person’s needs and home environment characteristics should be considered prior to hospital discharge. This is especially important as Sweden and many other countries increasingly favor community-based health and social care services provided in patients’ homes, rather than assisted living and institutional care (SOU 2020). In addition, we were surprised that we did not find a significant association between falls at home and accessibility problems. Future studies with larger sample sizes are needed to investigate this further.

The finding that living in a highly inaccessible home is associated with lower self-efficacy, recovery after stroke, perceived environmental support, and worse perceived health deserves attention. While further studies are needed to determine the causality of these correlations, it is not farfetched to believe that the physical home environment for poststroke patients is especially important as the home is part of an integrated care chain. Gefenaite and colleagues (2020) found that self-efficacy may play a role in whether accessibility problems in the home affect persons’ abilities to be active in their daily life. Given that self-efficacy is associated with many rehabilitation outcomes poststroke (Jones & Riazi, 2011; Szczepańska-Gieracha & Mazurek, 2020), it would be interesting to explore if self-efficacy moderates the effects of the housing situation.

By using the ICF, we could identify those activities that include hand and arm use and walking as most critical in relation to accessibility. Knowledge about common difficulties for specific populations is important as it can be used to inform design solutions in the home among other things. Many of the barriers (e.g., “no grab bar in the bathroom”) identified in this study that caused accessibility problems for the participants are easy to overcome. Even though 50% of participants were reliant on a walking aid and therefore, more vulnerable to demands of the physical environment, only six people reported to have had housing adaptations. This suggests an unmet need. In addition, our findings revealed that the refuse bin and letter box were difficult to reach and caused accessibility problems. Such barriers cannot be easily overcome through housing adaptations but needs attention of policy makers and urban planners at a societal level. For example, as has been noted also in a recent study about accessibility problems among persons with Parkinson’s disease (Andersson et al., 2022), the new standards of waste management in Sweden (e.g., sorting garbage by type) may not benefit people with functional limitations and should consequently be addressed at a societal level rather than through individual adaptations.

Barriers in the home environment are often regarded as negative and pose a potential risk for injuries such as falls. However, persons in the home can also be seen as active agents and, in this way, may change the environment to fit their needs. This, in turn, may generate feelings of empowerment. If the environment is not fixed, the users will change it. This line of reasoning can be linked to the notion of everyday design where users are seen as everyday designers who interact with the environment and make changes to it or use it differently to fit their needs (Wakkary & Maestri, 2008). We believe it is important for practitioners to acknowledge such dynamic interplay and not see only barriers but also consider the environment as a resource that can be used in the rehabilitation process.

The results demonstrating perceived participation in the planning of care and rehabilitation was low in our sample; this is not a unique but an alarming finding. Achieving participation of people with stroke has been reported to be a complex endeavor (Bratzke et al., 2015; Bunn et al., 2018). It is a serious concern that people who have experienced a life-threatening event do not feel involved in their continued care and rehabilitation. In our study, we specifically asked if the participants had been given the opportunity to discuss the environment at home. At the time of discharge from the hospital, few participants reported having had such an experience. However, for some, this experience changed for the better when they had returned home and met the ESD team. The findings highlight that the environment is still not fully incorporated as an essential factor to consider and integrate into the rehabilitation process across the continuum of care. In addition, ESD has not yet been fully implemented, and many patients poststroke do not benefit from such home rehabilitation models (SBU, 2015). The findings indicate that bodily function and medical aspects still have a central role in hospital discharge discussions. The physical home environment is seldom considered, and it might be easier to address environmental factors when the practitioners meet the person at home when they can observe the environment as the person moves around and performs daily activities (Kylén et al., 2021). However, a recent review of instruments aiming to measure patient participation in healthcare (Kylén et al., 2022) revealed that few reflected participation and environmental aspects. In fact, the authors did not find a single patient reported instrument that incorporated the physical home environment as part of patient participation. Further studies should be conducted to explore the potential causes of leaving the environment outside the person’s life situation.

### Study Limitations

This study has several limitations. The small sample size and cross-sectional design impedes the determination of causality. Consequently, longitudinal studies investigating the effect of the physical home environment on rehabilitation outcomes such as perceived health and self-efficacy poststroke is warranted. Nonetheless, while objective aspects of the physical home environment are important to consider, it also affects people’s well-being and daily activities through its meaning which is not easily captured using quantitative approaches.

We collected the data approximately 3 months poststroke; this implies that some participants experienced difficulty in retrospectively reporting their experiences of being involved in the planning of their care and rehabilitation, especially during hospital discharge. All participants suffered mild/moderate stroke and the findings, therefore, cannot be transferred to persons with severe stroke and aphasia. Our study was conducted in an ESD context and persons with severe stroke are seldom discharged to home with ESD as they need more advanced rehabilitation support, often inpatient. However, given that accessibility is the outcome of a person’s level of functioning and the design of the physical environment, it is not farfetched to believe that rehabilitation outcomes are worse for persons with severe stroke; this calls for further research using inclusive and flexible research methods (e.g., photo voice, observations). In addition, today, there are technical methods such as sensors that objectively can monitor postural stability and level of mobility inside and outside the home. Such methods are promising and can be used in future research. Another limitation concern how housing adaptations were identified in the present study. Housing adaptations may not always be obvious to an observer, while participants may not consider small changes to the environment as an adaptation.

## Conclusions

This explorative study has generated new knowledge highlighting that many face problems adapting their home environments poststroke and that accessibility problems may be associated with worse health, recovery after stroke, self-efficacy, and perceived environmental support for persons who are rehabilitating at home. Unfortunately, our study adds to the growing literature demonstrating that persons with complex health conditions do not often experience involvement in the planning of their continued care and rehabilitation. In particular, we highlighted that there is still much left to do with respect to raising awareness among healthcare practitioners about the importance of considering the physical home environment in the rehabilitation process. The findings could be used by architects, urban planners, policy makers, and healthcare practitioners to address unmet needs and recommend more effective housing planning and inclusive environments. Further studies are needed to determine causality of identified associations.

## Implications for Practice

- Healthcare practitioners should consider the unmet needs of persons with stroke when planning for rehabilitation which includes the physical environment.
- The article shows that the activities most restricted by environmental barriers in the home concerned hand and arm use, this is new knowledge that can inform healthcare practitioners when planning interventions and home adaptations.
- Many patients face housing accessibility problems that may hinder their recovery, yet the findings show that few were given the possibilities of discussing the physical environment with healthcare staff.
- Architects should focus on creating more inclusive and accessible housing environments to improve the quality of life for persons with stroke and thus support their rehabilitation process.
- Collaboration between healthcare practitioners, architects, and planners is essential to create supportive environments and thus better rehabilitation outcomes for persons with stroke.
